# RACK1 is involved in endothelial barrier regulation via its two novel interacting partners

**DOI:** 10.1186/1478-811X-11-2

**Published:** 2013-01-11

**Authors:** Anita Boratkó, Pál Gergely, Csilla Csortos

**Affiliations:** 1 Department of Medical Chemistry, University of Debrecen Medical and Health Science Center, Egyetem tér 1, Debrecen, H 4032, Hungary

**Keywords:** Endothelial cell, Prenylation, RACK1, TIMAP

## Abstract

**Background:**

RACK1, receptor for activated protein kinase C, serves as an anchor in multiple signaling pathways. TIMAP, TGF-β inhibited membrane-associated protein, is most abundant in endothelial cells with a regulatory effect on the endothelial barrier function. The interaction of TIMAP with protein phosphatase 1 (PP1cδ) was characterized, yet little is known about its further partners.

**Results:**

We identified two novel interacting partners of RACK1, namely, TGF-β inhibited membrane-associated protein, TIMAP, and farnesyl transferase. TIMAP is most abundant in endothelial cells where it is involved in the regulation of the barrier function. WD1-4 repeats of RACK1 were identified as critical regions of the interaction both with TIMAP and farnesyl transferase. Phosphorylation of TIMAP by activation of the cAMP/PKA pathway reduced the amount of TIMAP-RACK1 complex and enhanced translocation of TIMAP to the cell membrane in vascular endothelial cells. However, both membrane localization of TIMAP and transendothelial resistance were attenuated after RACK1 depletion. Farnesyl transferase, the enzyme responsible for prenylation and consequent membrane localization of TIMAP, is present in the RACK1-TIMAP complex in control cells, but it does not co-immunoprecipitate with TIMAP after RACK1 depletion.

**Conclusions:**

Transient parallel linkage of TIMAP and farnesyl transferase to RACK1 could ensure prenylation and transport of TIMAP to the plasma membrane where it may attend in maintaining the endothelial barrier as a phosphatase regulator.

## Background

The vascular endothelium functions as a semi-permeable barrier between blood and the interstitium. Endothelial cell (EC) barrier regulation is under intense investigation, since EC barrier dysfunction is a well-known feature of acute lung injury (ALI) and its more severe form, acute respiratory distress syndrome (ARDS) [1]. Barrier enhancing or protecting processes are not explored in details yet, however, several studies indicate the significance of dephosphorylation of key cytoskeletal/membrane associated targets by specific protein phosphatases [2]. TIMAP (TGF-β inhibited membrane-associated protein) protein has been considered as a member of the MYPT (myosin phosphatase targeting) family of the regulatory subunits of protein phosphatase 1 (PP1) based on its structural features [3]. TIMAP and MYPT3 are the most closely related members within the family. Both proteins contain ankyrin repeats, a PP1 catalytic subunit (PP1c) binding motif and a C-terminal prenylation motif; the latter one mediates their association with the plasma membrane [3]. A bipartite nuclear localization signal (NLS) is also present in TIMAP; accordingly, it was detected in the nucleus of vascular endothelial cells [4], but its significance is not known yet. In view of the fact that TIMAP mRNA synthesis is down-regulated by TGF-β1 [3], the transcriptional repression of TIMAP could be an important component of the TGF-β1 pathway, as well as apoptosis and endothelial barrier integrity. TIMAP is most abundant in endothelial cells, yet little is known about its exact function and its interacting partners. We have demonstrated specific protein-protein interaction between TIMAP and PP1cδ (a.k.a. PP1cβ); and the effect of TIMAP phosphorylation on this interaction was also characterized [4,5]. Studies made on TIMAP-depleted human pulmonary artery endothelial cell (HPAEC) monolayers indicated that TIMAP has a positive regulatory effect on the endothelial barrier function. In the absence of TIMAP the effects of barrier function compromising agents (thrombin and nocodazole) were enhanced and the effects of barrier function protecting agents (sphingosine-1-phosphate and ATP) were attenuated [4]. Our work revealed that the ERM (ezrin-radixin-moesin) proteins, which connect actin filaments to the plasma membrane, are interacting protein partners for TIMAP and they were identified as TIMAP-PP1c substrates [4,5]. We also showed the involvement of TIMAP in PKA-mediated ERM dephosphorylation as part of endothelial cell barrier protection by TIMAP [4,5].

The non-integrin laminin receptor 1 (LAMR1), which is involved in the regulation of cell motility, was also described to interact with TIMAP. They co-localize at the plasma membrane of endothelial cells and TIMAP regulates the dephosphorylation of LAMR1 by PP1c [6]. Several potential TIMAP-binding proteins were identified by bacterial two-hybrid screening. Among them are the cysteine and glycine rich protein 1 and the eukaryotic translation elongation factor 2, which are both involved in the organization of the actin cytoskeleton. However, these interactions in mammalian cells have not been verified yet [7].

In a hunt for new TIMAP-binding partners, we found that RACK1 (receptor of activated kinase 1, a.k.a. guanine nucleotide-binding protein subunit beta-2-like 1, GNB2L1, or human lung cancer oncogene 7 protein) interacts with TIMAP in pulmonary artery endothelial cells. RACK1 is a scaffolding/anchoring protein that contains seven Trp-Asp (WD) repeats predicted to form a seven-bladed propeller structure. Therefore RACK1 is capable to interact simultaneously with several signaling molecules [8], suggesting the possibility of multiple protein interaction surfaces. It has been named by its binding to activated PKC [9], but several other interacting partners were also identified [10-14]. Upon activation of PKC with PMA, RACK1 co-localizes with the Src tyrosine kinase at the plasma membrane [15]; and RACK1 functions as a substrate, as well as a binding partner and inhibitor of the kinase [15,16]. Recently, it was also demonstrated that RACK1 is a core component of the eukaryotic 40S ribosomal subunit [17-19], localized on the head region close to the mRNA exit channel, suggesting a physical link between the eukaryotic ribosome and cell signaling pathways *in vivo*[20]. In accord with its structural features, all these recent findings imply the involvement of RACK1 in numerous signaling pathways.

Here, we report and characterize the interaction of RACK1 with TIMAP, a protein involved in the regulation of endothelial barrier function, and with farnesyl transferase, the enzyme catalyzing prenylation of TIMAP in endothelial cells. We show the significance of these interactions in the prenylation of TIMAP, consequently in its traffic to the plasma membrane.

## Results

### RACK1 binds TIMAP-PP1c complex in endothelial cells

**Figure 1:**
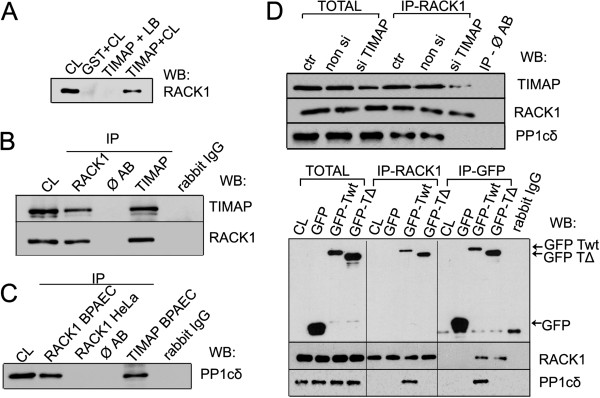
**RACK1 interacts with PP1cδ via TIMAP. **(**A**): Bacterially expressed glutathione S-transferase (GST) and GST-tagged wild-type TIMAP were loaded onto glutathione-Sepharose as described in Materials and Methods. After a washing step the resin samples were incubated with BPAEC lysate (CL) or cell lysis buffer (LB). Non-binding proteins were washed out and the bound proteins were eluted with 10 mM glutathion. Western blot probed with RACK1 specific antibody (**A**) of the endothelial cell lysate (CL) and the eluted fractions after the pull-down are shown. (**B**,**C**): RACK1 or TIMAP was immunoprecipitated from lysates of BPAEC (**B**) and BPAEC or HeLa (*C*) cells as described in Materials and Methods. IP complexes were probed for TIMAP and RACK1 (*B*) or PP1cδ (**C**). CL: cell lysate, Ø AB: control of IP from BPAEC without the addition of antibody. (**D**,**E**): RACK1 or GFP was immunoprecipitated from non transfected (CL), non-siRNA, TIMAP specific siRNA (si TIMAP), pEGFP-C1 (GFP), pEGFP-C1 TIMAP WT (GFP-Twt) or pEGFP-C1 TIMAPΔpp1c (GFP-TΔ) transfected HeLa cell lysates. IP complexes were probed for GFP, RACK1 or PP1cδ.

To identify new interacting partners of TIMAP in endothelial cells, GST pull-down assay was performed. BPAEC lysate was incubated with GST-tagged recombinant full length TIMAP protein immobilized on glutathione Sepharose. GST protein incubated with the cell lysate and GST-TIMAP incubated with the lysis buffer were used as negative controls. After the necessary washing steps the eluted proteins were separated on SDS-PAGE stained with BlueSilver solution and the pattern of the protein bands in the three samples were compared. An extra band at about 34 kDa was identified in the sample of GST-TIMAP incubated with BPAEC lysate (Additional file 1: Figure S1). It was cut from the gel and was further analyzed by LC-MS/MS (Dr. Janáky, University of Szeged). Bovine RACK1 (Gene: GNB2L1, Accession number: P63243) was identified using Swissprot and Uniprot TREMBL database. This result was confirmed by Western blot analysis of the pull-down samples using anti-RACK1 antibody (Figure 1A). Specific band of RACK1 can be seen in the total cell lysate (positive control) and in the GST-TIMAP sample which was incubated with the cell lysate, however, no RACK1 signal was detectable in the two negative control samples suggesting a specific interaction between TIMAP and RACK1.

Immunoprecipitation experiments were utilized to verify the interaction of the endogenous proteins in endothelial cells. RACK1 was shown to be present in the immunoprecipitate (IP) of TIMAP and vice versa, TIMAP co-immunoprecipitated with RACK1 (Figure 1B).

Since we have shown earlier that TIMAP has a strong interaction with PP1cδ [4,5], the presence of PP1cδ in the TIMAP-RACK1 complexes were also examined. As it was expected, the phosphatase was present in both the RACK1 and the TIMAP IP complexes (Figure 1C). To test whether RACK1 binds PP1c directly without the attendance of TIMAP, immunoprecipitation was made with RACK1 antibody from HeLa cells that do not express endogenous TIMAP [3]. No interaction was detected between RACK1 and PP1cδ in HeLa cells (Figure 1C), therefore one may conclude that RACK1 interacts with PP1cδ via TIMAP. To further verify this result, RACK1 was immunoprecipitated from control, non-siRNA and TIMAP specific siRNA transfected EC. PP1cδ was not detected in the RACK1 IP from TIMAP depleted cells (Figure 1D). In addition, mammalian constructs were created to express recombinant wild type and truncated TIMAP. The truncated form does not contain the PP1c binding motif, as the first 68 amino acids are deleted; consequently it is expected not to bind the phosphatase. HeLa were transfected with empty pEGFP, wild type TIMAP/pEGFP (GFP-TIMAP wt) or truncated TIMAP/pEGFP (GFP-TIMAP ΔPP1c) plasmids and GFP or the endogenous RACK1 were immunoprecipitated. The IP complexes were probed for RACK1, GFP and PP1cδ (Figure 1E). Although HeLa contains PP1cδ, the phosphatase was not present in the RACK1 IP except in wild type TIMAP over-expressing HeLa. Moreover, PP1cδ was not detectable with the truncated form of TIMAP in the RACK1-GFP-TIMAP ΔPP1c complex, only in the RACK1-GFP-TIMAP wt complex. These results imply that PP1cδ is present in the latter complex due to its interaction with TIMAP, but there is no direct binding between PP1cδ and RACK1; furthermore, the presence of this phosphatase is not a requirement for TIMAP-RACK1 interaction.

### Mapping the TIMAP-RACK1 interaction domains

**Figure 2:**
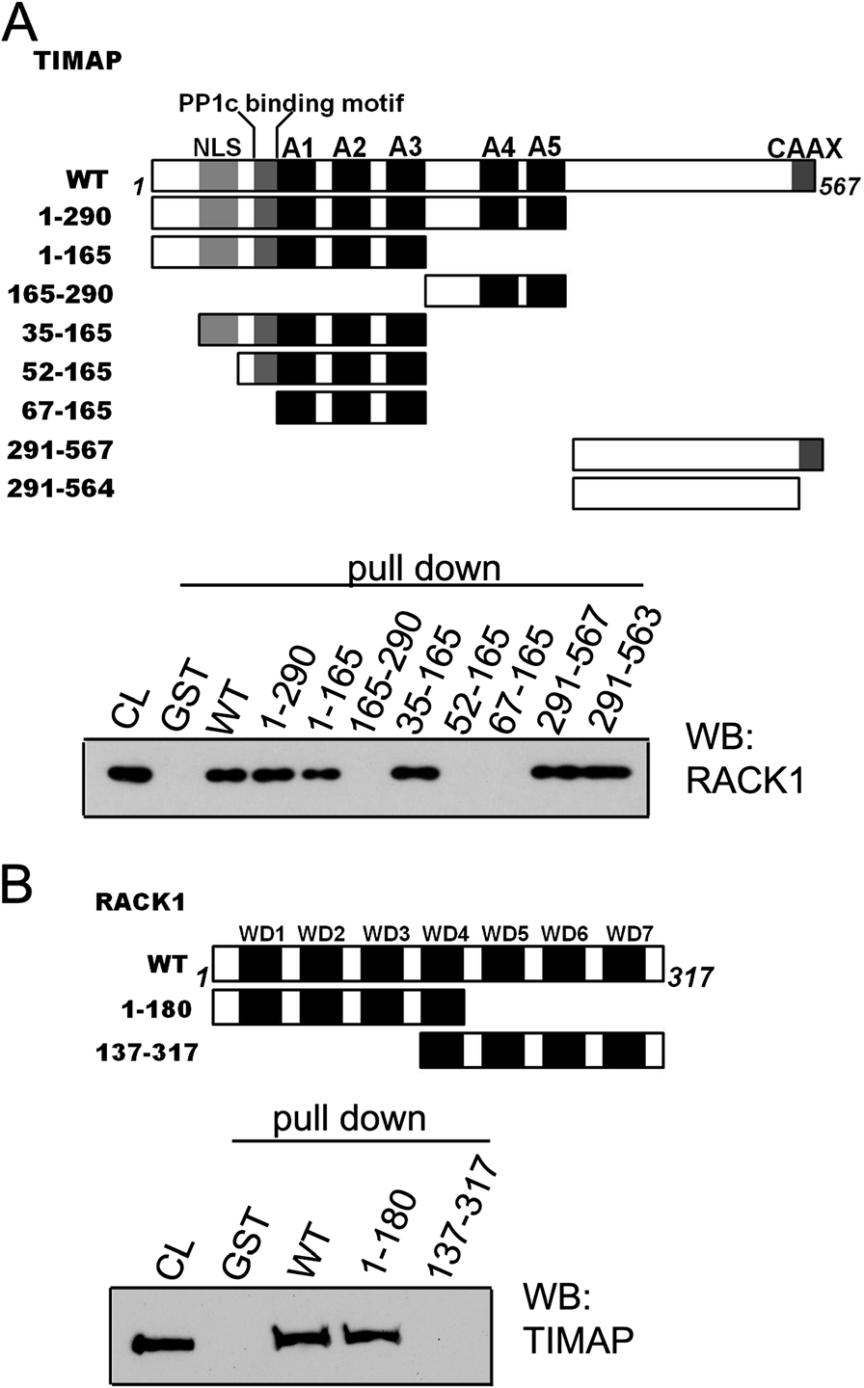
**Domain mapping of TIMAP-RACK1 interaction. **(**A**): GST-TIMAP pull-down of endogenous RACK1. GST, recombinant GST-TIMAP WT or additional GST-TIMAP fragments (depicted in the upper part of panel ***A***) were loaded onto glutathione-Sepharose as described in Materials and Methods. The immobilized protein samples were incubated with BPAEC lysate. Western blot of the pull-down eluates probed with anti-RACK1 antibody is shown. CL: total cell lysate. (**B**): GST-RACK1 pull-down of endogenous TIMAP. GST, recombinant RACK1 WT or GST-RACK1 fragments (depicted in the upper part of panel ***B***) were tested in pull-down assay. The immobilized samples were incubated with BPAEC lysate. Western blot of the pull-down eluates was probed with anti-TIMAP antibody. Representative data of at least 3 independent experiments are shown.

Several deletion mutants of TIMAP were created to identify its domains concerned in the RACK1 interaction (Figure 2A). The interface between bacterially expressed TIMAP and endogenous RACK1 was mapped by GST pull-down assay. Surprisingly, RACK1 was able to bind both to the N-terminal region (TIMAP 1–290) of TIMAP containing the nuclear localization signal (NLS), the PP1c-binding motif and the five ANK repeats, as well as to the C-terminal region (TIMAP 291–567) with the earlier identified PKA and GSK3β phosphorylation sites [21] and the C-terminal CAAX prenylation motif. The latter was excluded as a significant region of TIMAP in the interaction, since the C-terminal fragment missing the CAAX box (TIMAP 291–563) did not bind less RACK1 than TIMAP 291–567. To further specify the interacting region in the N-terminal section of the protein, additional shorter recombinants were tested. When the N-terminal fragment was shortened (TIMAP 1–165) we still could detect binding. The mutants containing only ANK4-5 and a region with unidentified function (TIMAP 165–290) or ANK1-3 (TIMAP 67–165) did not bind to RACK1. Therefore it was concluded that none of the ANK repeats are concerned. The very N-terminal region of TIMAP (spanning amino acids 1–34) do not affect the binding either. The short region of the potential NLS, however, appeared to be essential by the comparison of the binding ability of TIMAP 35–165 and TIMAP 52–165 to endogenous RACK1 as the only difference between these two fragments is the presence or absence of the NLS motif, respectively.

The β-propeller structure of RACK1 due to its seven WD repeats offers multiple docking sites for several interactions. The association of native TIMAP to bacterially expressed full length GST-RACK1, N-terminal (1–180) or C-terminal (137–317) GST-RACK1 truncated forms were studied in GST pull-down assays (Figure 2B). Our results clearly indicate that only the N-terminal half of RACK1 is involved in the RACK1-TIMAP interaction.

### Forskolin treatment attenuates the RACK1- TIMAP interaction

RACK1 (reviewed in [22]) and TIMAP [5,21] are recognized to be related to several kinases, and upon the activation of certain kinases changes in their protein-protein interactions were described. Namely, PKCs and RACK1 mutually influence each other, but RACK1 may participate in the cAMP/PKA pathway as well [10,12,23]. Recent results indicate that TIMAP is a target for PKA-primed GSK-3β mediated phosphorylation on sites Ser337 and Ser333, respectively [5,21]. Thus we next tested the effect of the activation of PKC and PKA on the TIMAP-RACK1 interaction challenging EC with PMA and forskolin, respectively.

**Figure 3:**
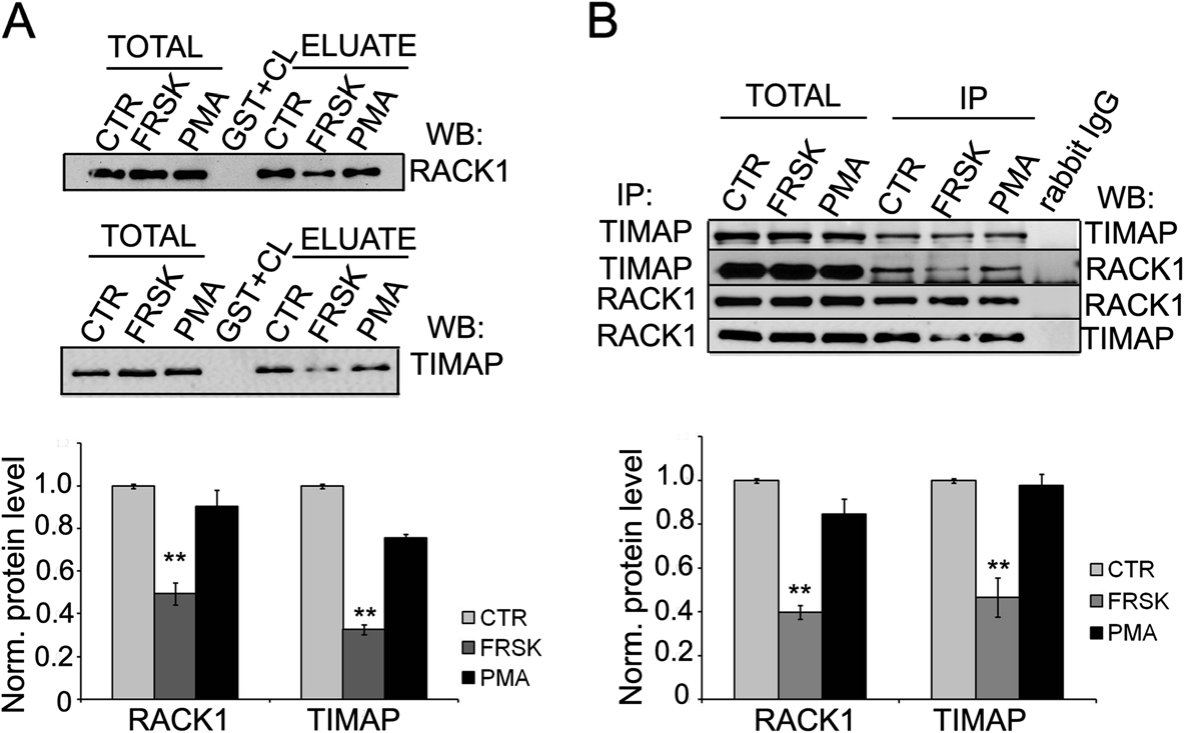
**TIMAP-RACK1 interaction is attenuated by the cAMP/PKA pathway. **(**A**) GST, full-length GST-TIMAP (upper part) or GST-RACK1 (lower part) were immobilized on glutathione-Sepharose and incubated with cell lysates of non treated (ctr), forskolin (50 μM for 30 min) (FRSK) or PMA (1 μM for 30 min) treated BPAEC. The eluted proteins were tested by Western blot using anti-RACK1 and anti-TIMAP antibodies. (**B**) Endogenous TIMAP or RACK1 was immunoprecipitated from BPAEC lysates after the same treatments described for panel *A*. IP complexes were probed for TIMAP and RACK1. Shown are representative data of means ± SE from at least 3 independent experiments. Protein levels were quantified by densitometric analysis. Eluted proteins were normalized against total protein levels.

The attenuative or restorative consequences of PMA or forskolin treatment on the interaction were established by GST pull-down assays first. Equal amounts of bacterially expressed GST-TIMAP or GST-RACK1 were loaded onto glutation Sepharose 4B as described in Materials and Methods and untreated, forskolin or PMA challenged endothelial cell lysates were added to the resin. Bound proteins in the eluates were analyzed by Western blot (Figure 3A). The amount of RACK1-TIMAP complex was considerably lower after the activation of the cAMP/PKA pathway (forskolin), on the other hand, PMA treatment of EC had no significant effect. These findings were further strengthened with endogenous proteins only, when IP complexes of RACK1 and TIMAP drawn from EC after the same treatments were analyzed by Western blot (Figure 3B). Truncated wild type, S333A/S337A phosphorylation deficient, and S333D/S337D phosphomimic mutants of a TIMAP fragment spanning amino acids 331–567 were overexpressed in *E*. *coli* and were utilized in pull-down experiments. Consistent with the above described findings, the amount of RACK1 bound to the phospomimic TIMAP fragment was decreased compared to the amount of RACK1 bound to wild type TIMAP or the phosphorylation deficient fragment (Additional file 2: Figure S2). These data suggest that the phosphorylation state of TIMAP may be an important factor in its interaction with RACK1.

### Activation of the cAMP/PKA pathway affects localization of TIMAP

**Figure 4:**
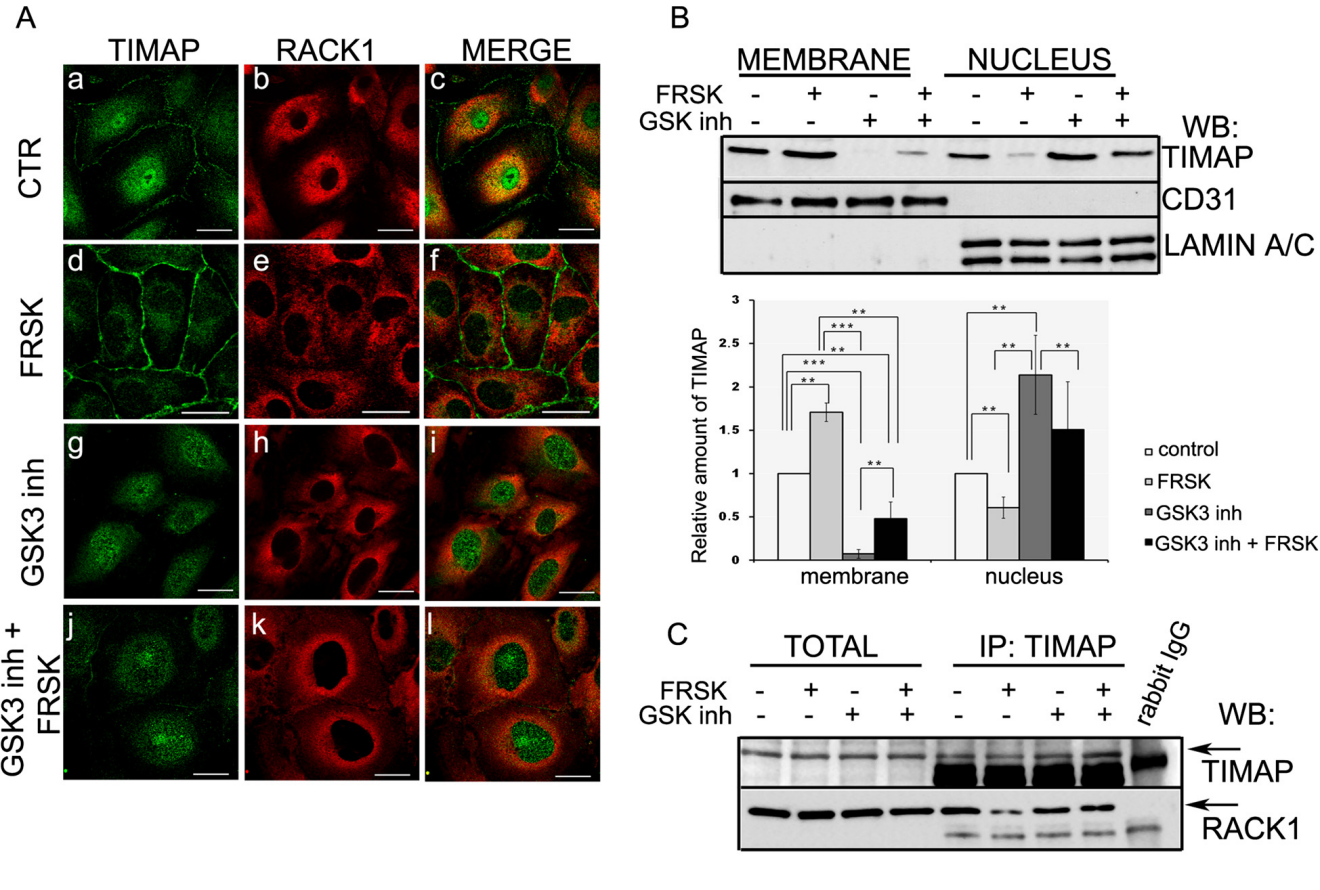
**GSK3β inhibitor results in loss of membrane localized TIMAP. **(**A**) Immunofluorescence staining of confluent HPAEC without (a-c) (CTR), or with various treatments as follows: 50 μM forskolin (FRSK) for 30 min (d-f); 20 μM GSK3β inhibitor, AR-A014418, (GSK3 inh) for 4 hrs (g-i); or 20 μM GSK3β inhibitor for 4 hrs followed by 50 μM forskolin for 30 min (j-l) using anti-TIMAP (a,d,g,j: green) and anti-RACK1 (b,e,h,k: red) primary antibodies was performed. Representative data of at least three independent experiments are shown. Scale bars: 100 μm. (**B**) Subcellular fractionations of HPAEC cells after the same set of treatments as listed in panel (*A*) were made as described in Materials and Methods. The fractions were analyzed with anti-TIMAP, anti-CD31 as membrane, anti-lamin A/C as nuclear and anti-β-tubulin (not shown) as cytoplasmic marker antibody. Shown are representative data of at least 3 independent experiments. Quantitative analysis of TIMAP signals is also shown. CD31 or lamin A/C bands were used for protein level normalization. The results are presented as means ± SE from 3 independent experiments. Statistical analysis was done with Student’s t-test. Significant changes are indicated by asterisks; * (P < 0.05), ** (P < 0.01), or *** (P < 0.001). (**C**) TIMAP was immunoprecipitated from HPAEC after the same set of treatments as listed in panel (*A*). Total cell lysates and the IP complexes were probed for TIMAP and RACK1. Additional bands in IP samples correspond to IgG.

TIMAP localizes to the cell membrane and it is also present in the nucleus and in the cytoplasm surrounding the nucleus in HPAEC monolayer [4]. We investigated whether the RACK1-TIMAP complex formation has any effect on the subcellular localization of TIMAP. To modulate the interaction, HPAEC monolayers were subjected to agents affecting the phosphorylation level of TIMAP and the subcellular localization was detected by immunofluorescence studies of the monolayers or by Western blot of subcellular fractions (Figure 4A,B). Confocal images on Figure 4A show that the applied effectors did not change the cytoplasmic localization of RACK1 (Figure 4A b,e,h,k). On the other hand, upon forskolin treatment, the amount of nuclear TIMAP decreased parallel with its more pronounced appearance in the cell membrane (Figure 4A d) compared to the untreated sample (Figure 4A a). When cells were pretreated with a PKA inhibitor, H89, no translocation of TIMAP to the cell membrane was observed upon forskolin challenge, proving the involvement of PKA activity (Additional file 3: Figure S3). Since PKA phosphorylation of TIMAP on Ser337 primes its GSK3β phosphorylation on Ser333 [5,21], AR-A014418, a selective GSK-3β inhibitor [24], was employed alone or as pretreatment before addition of forskolin to prevent PKA primed phosphorylation of TIMAP by GSK-3β. Without forskolin, no TIMAP was detected in the plasma membrane when GSK-3β was inhibited (Figure 4A g); also, the effect of forskolin was strongly attenuated in the presence of AR-A014418 (Figure 4A j). Merged images indicate co-localization of RACK1 and TIMAP in the region of cytoplasm that is rather close to the nucleus in control and GSK-3β inhibited cells cells (Figure 4A c,i,l), but co-localization was not detectable in the cells treated exclusively with forskolin (Figure 4A f).

Membrane and nuclear fractions of HPAEC were isolated by cell fractionation as described in Materials and Methods and the amount of TIMAP in the fractions was detected by Western blot (Figure 4B). Parallel with the results of the immunofluorescent staining, the amount of TIMAP increased in the membrane fraction after forskolin, but it was significantly lowered in the presence of GSK-3β inhibitor compared to the control. Forskolin challenge in GSK-3β inhibited cells caused significant increase in the TIMAP level in the membrane fraction compared to the extremely faint signal found in the same fraction of cells treated only with the kinase inhibitor. Furthermore, Western blot analysis of the nuclear fractions, as expected, demonstrated opposite trends for the changes. The least amount of TIMAP was detected in the nuclear fraction of the forskolin treated cells, while inhibition of GSK-3β caused a large increase, however, forskolin significantly moderated this effect of the inhibitor.

There was no significant change in the amount of RACK1 in TIMAP IP of GSK-3β inhibited cells compared to control. As expected, less RACK1 was associated to TIMAP after forskolin challenge, but no effect of forskolin was detectable after AR-A014418 pretreatment of EC (Figure 4C). These results suggest that interaction of TIMAP with RACK1 may exist in the cytoplasm of EC and it is affected by double phosphorylation (PKA and GSK-3β) of TIMAP.

### Effect of RACK1 depletion on TIMAP

**Figure 5:**
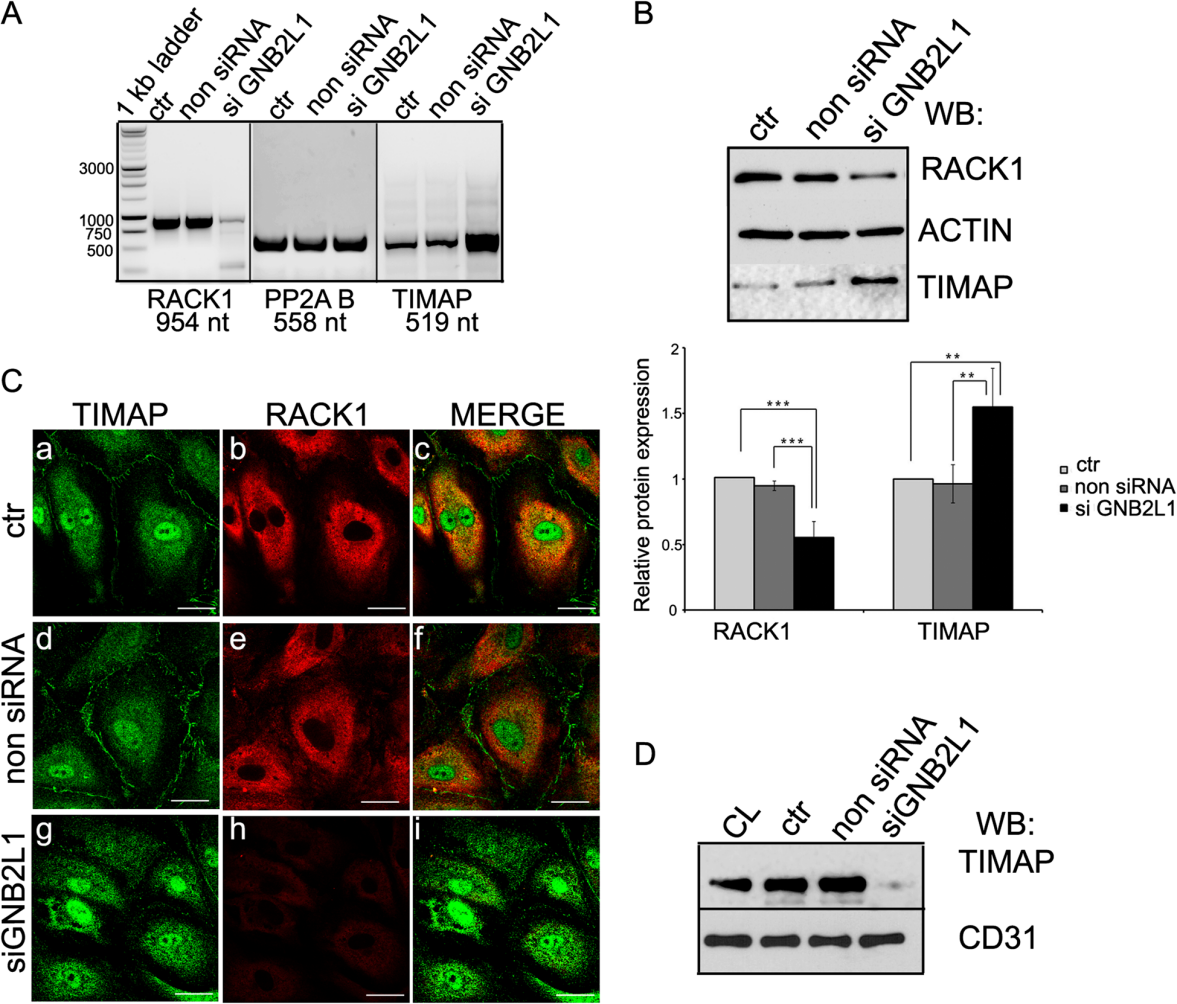
**RACK1 depletion modulates mRNA and protein levels of TIMAP and affects the membrane localization of TIMAP. **(**A**) HPAEC grown on 6-well plate was transfected with small interfering RNA (siRNA) as described in Materials and Methods. Total RNA was isolated from non transfected (ctr), non silencing RNA, or RACK1 specific siRNA (siGBN2L1) transfected cells and analyzed by RT-PCR using specific primer pairs for RACK1, PP2A B (irrelevant control) or TIMAP. (**B**) Western blot analysis of non transfected (ctr), non silencing RNA, or siGBN2L1 transfected cells using RACK1, TIMAP and actin specific antibodies. Actin was tested as loading control. The amount of RACK1 or TIMAP signal was expressed as ratio of RACK1/TIMAP:β-actin signal density. The error bars correspond to SE from 3 independent transfections. Statistical analysis was done with Student’s t-test. Significant changes are indicated by asterisks; ** (P < 0.01), or *** (P < 0.001). (**C**) Immunofluorescence staining of confluent non transfected (ctr) (a-c), non silencing RNA (d-f) or siGBN2L1 treated (g-i) HPAEC using anti-TIMAP (a,d,g: green) and anti-RACK1 (b,e,h: red) primary antibodies is presented. Scale bars: 100 μm. (**D**) Membrane fractions of non transfected (ctr), non silencing RNA or siGBN2L1 transfected HPAEC were isolated as described in Materials and Methods. Total cell lysate (CL) was also loaded as control. The fractions were analyzed with anti-TIMAP and anti-CD31 antibodies. Shown are representative data of at least 3 independent experiments.

RACK1 was depleted in HPAEC cells using silencing RNA duplexes specific for RACK1 (GNB2L1). The efficiency of silencing was confirmed both at mRNA and protein level of RACK1 by RT-PCR and Western blot (Figure 5A,B). We detected about 70-80% and 50% decrease in the mRNA and protein level of RACK1, respectively, in the depleted cells compared to the control or non silencing RNA transfected cells. Primers for B55, regulatory subunit of protein phosphatase 2A, were used as endogenous control of RT-PCR. In the same set of experiments the mRNA and protein level of TIMAP were evaluated as well; interestingly, both increased in the RACK1 depleted HPAEC (Figure 5A,B). This observation may suggest that RACK1 could be involved in the regulation of TIMAP transcription, but elaboration of this assumption would require further examination.

Immunofluorescent staining and Western blot analysis of membrane fraction of RACK1 siRNA transfected HPAEC revealed loss of TIMAP in the plasma membrane (Figure 5C,D). This offers another plausible interpretation of the increased amount of TIMAP, namely, RACK1 silenced cells simply try to compensate for the lowered membrane localized TIMAP.

**Figure 6:**
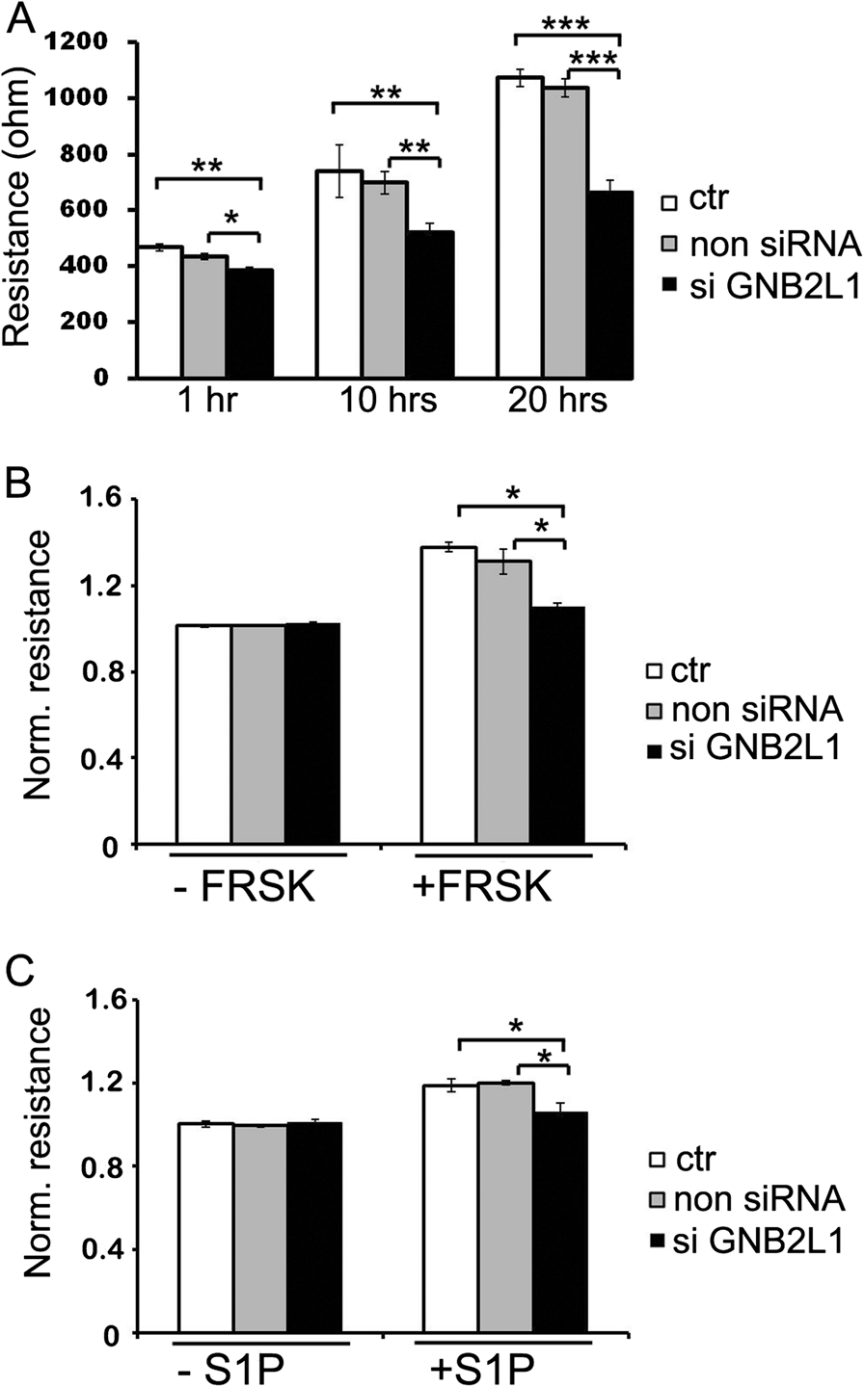
**RACK1 depletion attenuates EC barrier function. **Non transfected (ctr), non silencing or RACK1 specific silencing RNA transfected HPAEC were plated (5 x 10^5^ cells/well) onto two 8W10E arrays 48 h post-transfection. (**A**): The initial resistance values at the beginning of the measurement were about 300 Ω and the impedance was measured for 20 hrs after the seeding. (**B**,**C**): After overnight incubation and basal TER monitoring (900–1200 Ω), HPAEC monolayers were treated with 50 μM forskolin (**B**) or 1 μM sphingosine 1-phosphate (S1P) (**C**). Relative resistances that were detected at the time of maximal TER increase of forskolin/S1P treated cells are shown for each sample. The results are presented as means ± SEM at least of four chambers for each treatment.

Next it was tested whether the barrier function of EC monolayers could be affected by RACK1 depletion as a consequence of loss of TIMAP in the plasma membrane. Endothelial barrier formation (attachment and spreading of EC) of control and RACK1 depleted HPAEC were followed by ECIS measurement (Figure 6A). The number of control and siRNA transfected cells inoculated in ECIS wells was identical; also there was no notable difference in the cell density of the samples and no dead cells were observed in the wells after the ECIS measurements. The impedance values measured at 1 h after the start of the measurement were significantly lower for the RACK1 silenced sample. During the 20 hours of the experiment, this difference became more pronounced, implying that the formation of endothelial barrier was damaged in the absence of RACK1. In addition, the effect of forskolin and sphingosin 1-phosphate, two barrier enhancing agonists, was strongly attenuated in RACK1 depleted EC (Figure 6B,C).

### RACK1 aids farnesylation/membrane transport of TIMAP

**Figure 7:**
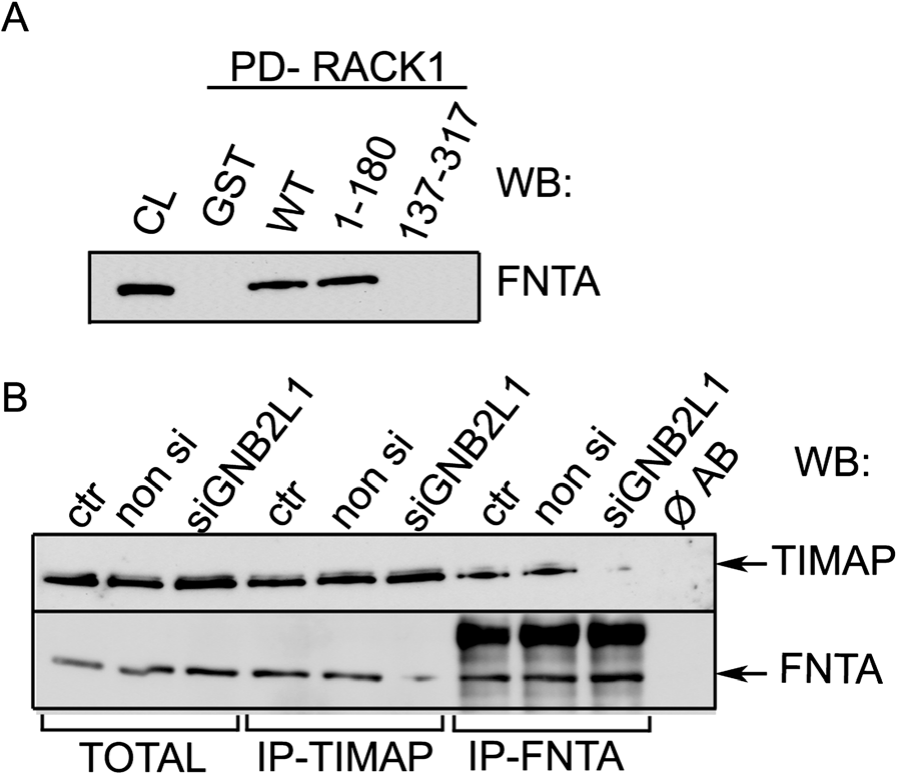
**TIMAP interacts with farnesyl transferase via RACK1. **(**A**) GST-RACK1 pull-down of endogenous farnesyl transferase (FNTA). GST, recombinant RACK1 WT or GST-RACK1 fragments were tested in pull-down assay. The immobilized samples were incubated with BPAEC lysate. Western blot of the pull-down eluates was probed with anti-FNTA antibody. (**B**) TIMAP or FNTA was immunoprecipitated from non transfected (ctr), non silencing RNA or siGBN2L1 transfected HPAEC. Total cell lysates and the IP complexes were probed for TIMAP and FNTA. Additional bands in IP samples correspond to IgG.

Since co-localization of TIMAP and RACK1 was not detected in our previous experiments in the cell membrane, we could exclude that RACK1 would be directly involved in the transport of TIMAP. Yet, we hypothesized that the prenylation of TIMAP leading to its movement to the plasma membrane may require the anchoring property of RACK1. Indeed, we detected interaction of farnesyl transferase with RACK1 in pull-down assay, the binding region in RACK1 is in the N-terminal WD1-4 region (Figure 7A). Most importantly, TIMAP and farnesyl transferase co-immunoprecipitated from HPAEC with normal RACK1 level only, but not from RACK1 depleted cells (Figure 7B), suggesting a pivotal role of RACK1 in TIMAP prenylation.

## Discussion

Vascular EC barrier integrity is critical to tissue and organ function [1,25]. The uniquely high expression of TIMAP protein in endothelial cells [3] implies its significance in fundamental activities of this cell type. In fact, our previous findings indicated its involvement in the regulation of endothelial cell barrier function [4]. Still, only a few of its protein interactions were identified [5-7]. In a search for further partners of TIMAP we recognized and proved by different methods that TIMAP binds the adaptor protein, RACK1. This work was focused on the characterization of this novel interaction.

RACK1 is known as a scaffolding protein which belongs to the WD-repeat containing proteins. It seems that RACK1 has no preference for a common structural feature in its binding partners. Among the RACK1 interacting proteins some contain Src homology (SH2) domains [14], pleckstrin homology (PH) domains like dynamin [23,26], C2 and V5 domains in PKCs [27,28] or PDZ domains [29]; but there is also example for a whole specific structural conformation requirement on the partner’s side [30]. Our results indicate that RACK1 binds to the NLS region at the N-terminal of TIMAP, but there is/are further association site(s) within the C-terminal half region of TIMAP suggesting a more complex surface for the interaction. It should be noted that the PKA/GSK-3β phosphorylation sites, Ser337/333 of TIMAP are present in this region. Moreover, we showed that the site/region in RACK1 responsible for TIMAP binding is within the N-terminal half (WD 1–4) of the protein. RACK1 forms homodimers via the fourth WD repeat [31], therefore both the N- and C-terminal mutants tested were designed to contain WD 4 as described by others [32]. Thus the binding region can be further narrowed to WD 1–3, but elucidation of the exact binding sites in TIMAP and RACK1 requires additional research. Another PP1 related protein, CPI17 (PKC potentiated PP1 inhibitor), was recognized as binding partner of RACK1 by yeast two-hybrid screening [33]. Interestingly, binding of the dimer form (catalytic and scaffolding subunit) of PP2A, another major Ser/Thr protein phosphatase, was shown to a C-terminal WD repeat in RACK1 [32,34]. The mutual or exclusive binding of the two subunits of PP2A was not resolved. We found that PP1cδ is present in the RACK1-TIMAP complex as TIMAP is its regulatory/targeting subunit, but does not bind directly to RACK1.

Although RACK1 and PKC are intimately related to each other (for review see [22]), that seems irrelevant in the TIMAP-RACK1 relation, as PKC activation of EC did not change their binding. On the other hand, activation of the cAMP/PKA pathway had significant effect not only on the interaction, but also on the localization of TIMAP. The second messenger cAMP is known as endothelial barrier stabilizer [35,36]. Upon cAMP/PKA activation of EC, we detected enrichment of TIMAP in the plasma membrane and its translocation from the nucleus. Since parallel translocation of RACK1 did not happen from the cytoplasm of EC either to the membrane or to the nucleus, this suggests that separate signaling pathways regulating nuclear export and membrane traffic of TIMAP could be initiated simultaneously.

RACK1 is connected to the cAMP/PKA signaling pathway through its interaction with cAMP phosphodiesterases [12,37]. Similar to our results, in hippocampal neurons dissociation of RACK1 from its binding partner, Fyn kinase, occurs upon activation of the PKA pathway [38]. RACK1 translocated to the nucleus in glioma and neuroblastoma cell lines upon PKA activation by forskolin [39] to mediate the expression of a brain-derived neurotrophic factor. In contrast, no translocation of RACK1 upon forskolin treatment of EC was observed in our experiments. However, it was revealed recently that phosphorylation by PKA or sequential phosphorylation by PKA and GSK-3β only slightly modulated the binding of TIMAP to PP1cδ [5]. The dissociation constant of the complex was about the same, only the rate of dissociation decreased to a small extent. *In vitro* phosphatase assays indicated that double phosphorylated form of TIMAP allowed PP1c activity toward phospho-moesin substrate, but mono- or non-phosphorylated form of TIMAP inhibited the phosphatase. PKA/GSK-3β phosphorylation sites, Ser337/333 of TIMAP are present in the C-terminal region which was shown to bind RACK1, thus the phosphorylation of these side chains may affect not only the regulatory effect of TIMAP on PP1cδ, but the binding of TIMAP to RACK1 as well. The phosphorylation may directly impair the connection by inducing conformation change of TIMAP, or may initiate interactions with other binding partners leading to the loss of RACK1-TIMAP complex. Our results clearly demonstrate that significant loss in TIMAP-RACK1 complex follows PKA primed GSK-3β phosphorylation of TIMAP.

On the contrary, when the TIMAP-RACK1 interaction was diminished by depletion of RACK1, TIMAP was not found in the plasma membrane of the silenced cells suggesting a pivotal role of RACK1 in prenylation/membrane localization of TIMAP. Significance of RACK1 in membrane localization of a Vang protein was also recognized by RACK1 knockdown recently [40]. Prenylation of TIMAP at the C-terminal CAAX box [3] by farnesyl transferase [41] is required for its membrane localization. Eventually, deficiency of membrane anchored TIMAP may be the result of the lack of its prenylation. Our results indicated that both TIMAP and farnesyl transferase bind to the N-terminal half of RACK1 and the interaction between TIMAP and farnesyl transferase was diminished in RACK1 depleted cells. These confirm the assumption of RACK1 being the anchoring surface for prenylation of TIMAP. Since TIMAP is involved in the regulation of EC barrier function [4], RACK1 should also be regarded as a participant in maintaining barrier integrity, through the regulation of TIMAP prenylation. The pulmonary vascular endothelium functions as a semi-selective barrier between blood and surrounding tissues and controls biological processes such as protein and fluid transport or inflammation. Endothelial barrier dysfunction is the primary cause of vascular leak and pulmonary edema in sepsis and is an essential component of angiogenesis, tumor metastasis, and atherosclerosis. Therefore, the maintenance of vascular EC barrier integrity may have profound clinical importance. In agreement with the conclusion that RACK1 is involved in the maintenance of barrier integrity, we found decelerated barrier formation in RACK1 depleted EC. Consistent with this, it was shown by others that RACK1 regulates cell adhesion [42], moreover, silencing of RACK1 inhibited cell proliferation and decreased migration and adhesion capability of carcinoma cells [43]. A recent paper [44] described the involvement of RACK1 in Gβγ-mediated adherens junction assembly in EC. They studied the function of Gβγ in re-annealing of adherens junctions after thrombin challenge/PAR activation. RACK1, normally bond to Gβγ, is released after thrombin and that triggers Fyn activation via the focal adhesion kinase. In RACK1 depleted cells, they detected slower recovery of TER after thrombin. Our TER measurement with thrombin treated RACK1 depleted cells showed the same result (not shown). Still, the positive effect of cAMP/PKA activation (forskolin treatment) on TER was significantly attenuated in RACK1 silenced EC. Moreover, sphingosin-1-phosphate, a well-known vascular stabilizer [45,46], had also failed to increase TER in RACK1 depleted cells. It seems, that RACK1 may be involved in multiple signaling pathways concerned in EC barrier regulation.

## Conclusions

**Figure 8:**
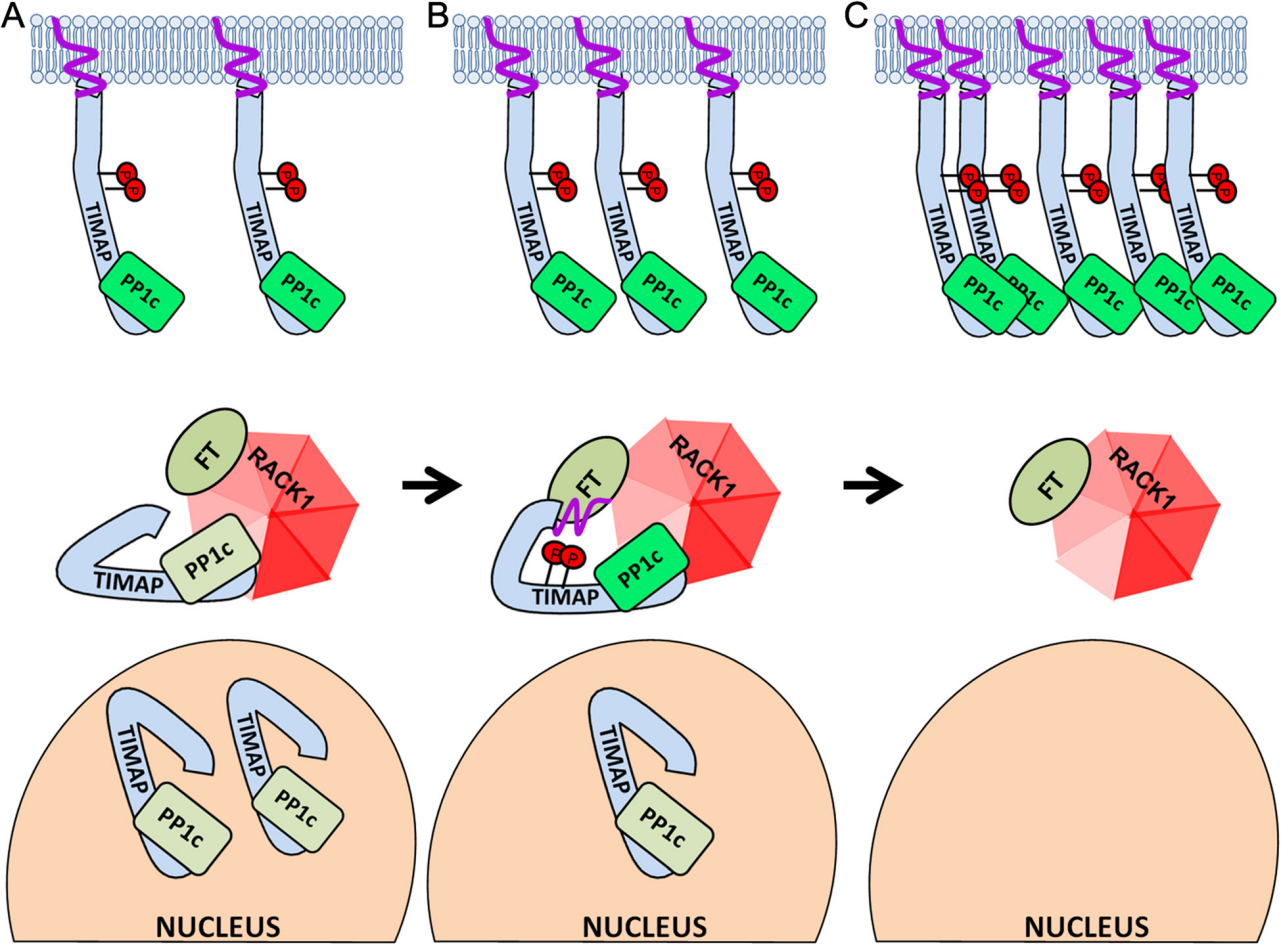
**Proposed model for prenylation and redistribution of TIMAP upon cAMP/PKA activation.** (**A**) In EC monolayer non-phosphorylated TIMAP is localized in the nucleus, also in the cytosol bound to RACK1, while prenylated and phosphorylated form of TIMAP is at the cell membrane. (**BC**) Upon PKA/GSK3β activation TIMAP in the RACK1 complex is phosphorylated resulting in conformation change, the C-terminal CAAX box becomes accessible for prenylation by farnesyl transferase (FT) bound also to RACK1. The prenylated TIMAP relocalizes to the cell membrane and RACK1 gets refilled with TIMAP from the nuclear pool. When that pool is exhausted, the amount of RACK1-TIMAP complex becomes low. Based on our previous result [5] PP1c is shown in complex with both non- and phosphorylated TIMAP, colors indicate different phosphatase activities of the complex. Representation of MYPT3 in [47] was used in the proposed conformational changes of TIMAP.

The anchoring protein RACK1 was recognized as a new TIMAP binding partner in EC and the regions of the interacting surfaces were identified. The interaction is transient, our results indicated that cAMP/PKA activation affected their binding and evoked a change in localization of TIMAP from the nucleus to the cell membrane. We propose that the phosphorylation of TIMAP pool bound to RACK1 in the cytosol may initiate a conformation change of TIMAP which facilitates its prenylation and translocation to the cell membrane. The cytosolic pool of TIMAP is “re-filled” from the nucleus, gets prenylated as well and moves to the plasma membrane (Figure 8). RACK1 supports this process by providing a simultaneous anchoring surface for TIMAP and farnesyl transferase. This ensures prenylation and subsequently membrane transport of TIMAP, where it may fulfill its barrier maintaining role as a PP1 regulatory protein.

## Methods

### Reagents

Materials were obtained from the following vendors: paraformaldehyde, dimethylsulfoxide, bovine serum albumin, forskolin, anti-FNTA antibody, AR-A014418, sphingosine 1-phosphate: Sigma (St Louis, MO), custom-made rabbit polyclonal anti-TIMAP antipeptide (NGDIRETRTDQENK) antibody: Zymed laboratories (San Francisco, CA), a kind gift from Dr. A. Verin, Georgia Health Science University, Augusta, GA; anti-RACK1 antibody: BD Transduction Laboratories (Heidelberg, Germany); anti-rabbit IgG HRP-linked and anti-mouse IgG HRP-linked secondary antibodies, CD31(PECAM-1) antibody: Cell Signaling Technology, Inc. (Beverly, MA); anti-PP1 delta antibody: Upstate Biotechnology (Lake Placid, NY); anti-lamin A/C (H-110) antibody: Santa Cruz Biotechnology, Inc. (Santa Cruz, CA); mouse anti-GFP antibody: Invitrogen Corporation (Carlsbad, CA); rabbit anti GFP: Merck Millipore (Billerica, MA); Alexa 488-, Alexa 594-conjugated secondary antibodies and ProLong Gold Antifade medium with DAPI: Molecular Probes (Eugene, OR), restriction enzymes, T4 DNA ligase: Thermo Scientific, Inc. (Vantaa, Finland); Protease Inhibitor Cocktail Set III: EMD Biosciences (San Diego, CA); pEGFP-C1, pGEX-4 T-2 and pGEX-4 T-3 vectors: Clontech Laboratories, Inc. (Mountain View, CA). Substances for cell culturing were from PAA (Austria). All other chemicals were obtained from Sigma (St Louis, MO).

### Cell cultures

Human Pulmonary Artery Endothelial Cells (HPAEC) (catalogue No: CC-2530) were obtained frozen at passage 3 (Lonza Group Ltd, Switzerland) and were cultured in EGM-2 Endothelial Cell Growth Medium-2 supplemented with 10% FBS and EGM-2 SingleQuots of Growth Factors. Cells were utilized at passages 5–7. Bovine pulmonary artery endothelial cells (BPAEC) (culture line-CCL 209) were obtained frozen at passage 8 (American Type Tissue Culture Collection, Rockville, MD), and were utilized at passages 15–20. Cells were maintained at 37°C in a humidified atmosphere of 5% CO_2_ and 95% air in MEM supplemented with 10% (v/v) heat inactivated fetal bovine serum, 1% sodium pyruvate, 0.1 mM MEM non-essential amino acids solution. HeLa cells (catalogue No: 93021013) were obtained frozen at passage 4 (ECACC, Salisbury, UK) and maintained in DMEM supplemented with 10% (v/v) FBS, 2 mM glutamine and 0.1 mM non-essential amino acids solution.

### SDS-PAGE and LC-MS/MS analysis

Proteins were resolved by SDS-PAGE and stained with Blue Silver solution [48]. Liquid Chromatography with Tandem Mass Spectrometry Detection was performed by Dr. Tamás Janáky at the University of Szeged, Faculty of Medicine, Department of Medical Chemistry. All samples were washed with 0.1 M NH_4_HCO_3_ and acetonitrile then digested with trypsin for 16 hours. The samples were dissolved in 0.1 M formic acid/H_2_O and aliquots (15 μL) were injected onto Waters NanoAcquity UPLC-QTOF trap. The retained materials were placed onto Waters BEH C18 trap (1.7 μm, 250×0.075 mm) and eluted (350 nL/min) with an increasing concentration of 0.1% formic acid/acetonitrile. Eluted peptides were analyzed by Data Dependent Aqusition and the three most abundant precursor ions were selected for MS/MS. Data were evaluated with ProteinLynx GlobalServer 2.4 software and Mascot 2.04 data browser.

### Preparation of TIMAP and RACK1 constructs

The coding region of wild-type TIMAP (NM_015568) was amplified by RT-PCR as described earlier [4]. Additional bacterial TIMAP constructs (the corresponding peptide regions are identified by the number of N- and C-terminal amino acids) were derived from the bacterial full length TIMAP construct and cloned into pGEX-4 T-3 vector using the following primer pairs. TIMAP 1–290: 5’- TGGGATCCATGGCCAGTCACGTGG-3’,  5’-AACTCGAGCTATGCACTGAGACTAGCTC-3’;TIMAP 1–165: 5’-TGGGATCCATGGCCAGTCACGTGG-3’, 5’- TTCTCGAGCTACGAGTTGACAGCAGGCA-3’; TIMAP 165–290: 5’- TAGGATCCGATGGGAACATGCCATATGA-3’, 5’- AACTCGAGCTATGCACTGAGACTAGCTC-3’; TIMAP 35–165: 5’- TGGGATCCAAGAAATGGGCACAGTACG-3’; 5’- TTCTCGAGCTACGAGTTGACAGCAGGCA-3’; TIMAP 52–165: 5’- TTGGATCCGAGCGGAAGCGCAGCA-3’, 5’- TTCTCGAGCTACGAGTTGACAGCAGGCA-3’; TIMAP 67–165: 5’- TTGGATCCGAGGCCAGCGTGGC-3’, 5’- TTCTCGAGCTACGAGTTGACAGCAGGCA-3’; TIMAP 291–567: 5’- TTGGATCCAGGACATCCATGGATGAGATG-3’, 5’- CGCTCGAGTCCTAGGAGATACGGCAAC-3’; TIMAP 291–564: 5’- TTGGATCCAGGACATCCATGGATGAGATG-3’, 5’- TTCTCGAGCTAACAGCCATGCACCTTCT-3’; TIMAP 331–567: 5’-TAGGATCCTCCTTGAGCCGGAGGACCTCCAG-3’, 5’- CGCTCGAGTCCTAGGAGATACGGCAAC-3’; TIMAP 331–567 S333A/S337A: 5’-TAGGATCCTCCTTGGCTCGGAGGACCGCTAG-3’, 5’- CGCTCGAGTCCTAGGAGATACGGCAAC-3’; TIMAP 331–567 S333D/S337D: 5’-TAGGATCCTCCTTGGACCGGAGGACCGACAG-3’, 5’- CGCTCGAGTCCTAGGAGATACGGCAAC-3’. Mammalian wild type and truncated form (does not contain the first 68 amino acids, including the PP1c binding motif) of TIMAP constructs were amplified using the following primer pairs and cloned into pEGFP-C1 vector. TIMAP WT: 5’-GGCTCGAGCTATGGCCAGTCACGTGGACCT-3’, 5’-CGCGGATCCCTAGGAGATACGGCAACAGCC-3’; TIMAP Δpp1c: 5’-GGCTCGAGCTATGAGCGTGGCCCTGCTGG-3’, 5’-CGCGGATCCCTAGGAGATACGGCAACAGCC-3’. Human cDNA prepared from HeLa cells by RT employing oligo dT primer was used for RACK1 (NM_006098.4) amplification with the following primers: RACK1 WT: 5’-GTGTCGACTATGACTGAGCAGATGACCCT-3’, 5’-AAGCGGCCGCCTAGCGTGTGCCAATGGT-3’; RACK1 1–180: 5’-GTGTCGACTATGACTGAGCAGATGACCCT-3’, 5’- AAGCGGCCGCCTAAGCCAGGTTCCATACCT-3’; RACK1 137–317: 5’-GTGTCGACTGTGTGCAAATACACTGTCCAG-3’, 5’-AAGCGGCCGCCTAGCGTGTGCCAATGGT-3’. All primers were synthesized by Integrated DNA Technologies (Coralville, IA). The DNA sequences of the constructs were confirmed by sequencing (Clinical Genomics Center, MHSC, RCMM, University of Debrecen).

### Bacterial expression and GST pull-down assay

Escherichia coli BL21 (DE3) transformed with pGEX-4 T-3 containing glutathione S-transferase (GST), pGEX-4 T3 containing TIMAP mutants or pGEX-4 T-2 containing RACK1 constructs were induced with 1 mM IPTG and grown at room temperature (RT) with shaking for 3 h. Cells were harvested by centrifugation, sonicated in lysis buffer (50 mM Tris–HCl (pH 7.5), 0.1% Tween 20, 0.2% 2-mercaptoethanol, protease inhibitors) and proteins were isolated by affinity chromatography on glutathione Sepharose 4B (GE Healthcare, Piscataway, NJ) according to the manufacturer’s protocol. BPAEC grown in 100-mm culture flasks were washed twice with 1X ice-cold PBS, scraped, and lysed in 600 μl lysis buffer. The lysates were incubated with GST or different GST-fused proteins coupled to glutathione Sepharose beads for 4 h at 4°C. The beads were washed three times with 1X PBS then the GST fusion proteins were eluted with 10 mM glutathione and were tested for interacting proteins by SDS-PAGE and Western blot.

### Transfection, siRNA silencing

HeLa cells were transfected with pEGFP-C1, pEGFP-C1 TIMAP WT or pEGFP-C1 TIMAP Δpp1c plasmids using Lipofectamine 2000 transfection reagents (Invitrogen Corporation (Carlsbad, CA), according to the manufacturer’s instructions. After 24 hours cells were washed with PBS and used for immunoprecipitation.

RACK1 (GNB2L1) and TIMAP (PPP1R16B) were silenced using 50 nM ON-TARGETplus SMARTpool siRNA (L-006876-00-0 HumanGNB2L1 and L-004065-00-0 HumanPP1R16B, respectively, Dharmacon) in complex with DharmaFECT-4 transfection reagent (Dharmacon) in serum-free medium. ON-TARGETplus siCONTROL nontargeting pool (D-001810-10-01-05; Dharmacon) was used as an irrelevant control. After 6 h the medium was changed to complete medium. Cells were further incubated for 48 hours.

### RT-PCR

Confirmation of RACK1 silencing was performed by RT-PCR. Total RNA was extracted from HPAEC using ZR RNA MicroPrep™ (Zymo Research Corporation, CA). cDNA was synthesized from 2 μg of total RNA using oligo-dT primer and M-MLV reverse transcriptase (Promega Corporation, USA). For PCR Phusion® High-Fidelity DNA Polymerase (Thermo Scientific, Inc.,Vantaa, Finland) was used. To monitor the amount of RACK1, TIMAP and PP2A B55 RNA, the following primers were used: RACK1 (GNB2L1): 5’-GTGTCGACTATGACTGAGCAGATGACCCT-3’, 5’-AAGCGGCCGCCTAGCGTGTGCCAATGGT-3’, TIMAP (PPP1RB16): 5’-TGGGATCCGAGAATAAGGACCCTAACC-3’, 5’- CGCTCGAGTCCTAGGAGATACGGCAAC-3’, PP2A B55 (PPP2R2A): 5’-CAGCACCTTCCAGAGCCA-3’, 5’-GGCAGATGCCCTCATGTC-3’.

### Immunofluorescence and microscopy

Cells were grown on glass coverslips, washed once with 1X PBS (137 mM NaCl, 2.7 mM KCl, 4.3 mM Na_2_HPO_4_, 1.47 mM KH_2_PO_4_, pH 7.4) and fixed with 3.7% paraformaldehyde in 1X PBS for 10 min at RT. Between each step, the cells were rinsed three times with 1X PBS. The cells were permeabilized with 0.5% Triton X-100 in PBS at RT for 15 min, blocked with 2% BSA in PBS for 30 min at RT, and incubated with primary, then with secondary antibodies diluted in blocking solution for 1 h at RT. Cover slips were rinsed and mounted in ProLong Gold Antifade medium. Confocal images were acquired with an Olympus Fluoview FV1000 confocal microscope using UPLSAPO 60× 1.35 NA oil immersion objective on an inverted microscope (Olympus IX81) at 25°C. Images were processed using FV10-ASW v1.5 software and further processed with PhotoShop Imaging software. Nonspecific binding of the secondary antibodies was checked in control experiments (not shown).

### Immunoprecipitation

BPAEC were grown in 100-mm tissue culture dishes, rinsed three times with 1x PBS and then collected and lysed with 600 μl of immunoprecipitation (IP) buffer (20 mM Tris HCl, pH 7.4, 150 mM NaCl, 2 mM EDTA, 2 mM sodium vanadate, 1% Nonidet P-40) containing protease inhibitors. The lysates were centrifuged with 10,000 g for 15 min at 4°C. To avoid nonspecific binding, the supernatants were precleared with 50 μl of protein G Sepharose (GE Healthcare, Piscataway, NJ) at 4°C for 3 h with end-over-end rotation. Protein G Sepharose was removed by centrifugation at 4°C for 10 min, and the supernatant was incubated with the appropriate volume of antibody at 4°C for 1 h and then with 50 μl of fresh protein G Sepharose at 4°C overnight with gentle rotation. The resin was washed three times with 300 μl of IP buffer and then resuspended in 150 μl of 1X SDS sample buffer, boiled, and microcentrifuged for 5 minutes. The supernatant was further analyzed by Western blot.

### Subcellular fractionation

Membrane fractions were isolated using ProteoJET™ Membrane Protein Extraction Kit and nuclear fractions were extracted by ProteoJET™ Cytoplasmic and Nuclear Protein Extraction Kit (Thermo Scientific, Inc.,Vantaa, Finland) according to the manufacturer’s protocol. The efficiency of fractionation was analyzed by immunoblotting using CD31 antibody as a membrane marker, lamin A/C antibody as a nuclear marker and β-tubulin antibody (not shown) as a cytoplasmic marker.

### Western blotting

Protein samples were separated by SDS-PAGE and transferred to 0.45 μm pore sized Hybond ECL Nitrocellulose Membrane (GE Healthcare, Piscataway, NJ). Western blots were imaged using an Alpha Innotech FluorChem® FC2 Imager or Kodak Medical X-ray Developer.

### ECIS measurements

ECIS (Electric cell-substrate impedance sensing) model Zθ, Applied BioPhysics Inc. (Troy, NY) was used to monitor transendothelial electric resistance [49] of control or transfected cells seeded on type 8W10E arrays.

### Statistical analysis

Statistical analysis was done with Student’s t-test. Significant changes are indicated by asterisks; * (P < 0.05), ** (P < 0.01), or *** (P < 0.001). Densitometric analyses of immunoblots were done by Image J software.

## Competing interests

The authors declare that they have no competing interests.

## Authors’ contributions

AB planned and carried out the experiments and made evaluations of results, drafted the manuscript. PG contributed in the evaluation of results and reagents/materials/analysis tools. CC planned the experiments and made evaluations of results, wrote the paper and contributed reagents/materials/analysis tools. All authors read and approved the final manuscript.

## Supplementary Material

Additional file 1 **Figure S1. **Detection of TIMAP-RACK1 interaction by pull-down. Bacterially expressed glutathione S-transferase (GST) and GST-tagged wild-type TIMAP were loaded onto glutathione-Sepharose as described in Materials and Methods. After a washing step the resin samples were incubated with BPAEC lysate (CL) or cell lysis buffer (LB). Non-binding proteins were washed out and the bound proteins were eluted with 10 mM glutathion. Blue silver staining of the endothelial cell lysate (CL) and the eluted fractions after the pull-down are shown. Red arrow points the band of a possible interacting protein (PI) appearing only in the TIMAP sample incubated with EC cell lysate (TIMAP + CL). That band was cut from the gel and was identified by LC-MS/MS as RACK1 which was confirmed by Western blot (Figure 1). Click here for file

Additional file 2 **Figure S2. **Phosphomimic mutation of TIMAP attenuates RACK1 binding. GST, recombinant GST-tagged truncated wild type, S333A/S337A, and S333D/S337D mutants of a TIMAP fragment (amino acids 331–567) were loaded onto glutathione-Sepharose as described in Materials and Methods. The immobilized protein samples were incubated with BPAEC lysate. Western blot of the pull-down eluates probed with anti-RACK1 antibody is shown. CL: cell lysate. Representative data of at least 3 independent experiments are shown. Click here for file

Additional file 3 **Figure S3. **Effect of PKA inhibition on the localization of TIMAP. Immunofluorescence staining of confluent HPAEC without (control), or with various treatments as follows: 10 μM H89 (PKA inhibitor) for 30 min; 50 μM forskolin for 30 min; or 10 μM H89 for 30 min followed by 50 μM forskolin for 30 min using anti-TIMAP antibody and Texas-Red Phalloidin. Scale bars: 100 μm. Click here for file
