# Supplementary figures and images for: RACK1 is involved in endothelial barrier regulation via its two novel interacting partners
Source: Cell Commun Signal. 2013 Jan 11;11:2. doi: 10.1186/1478-811X-11-2 (PMC3560227; doi:10.1186/1478-811X-11-2)

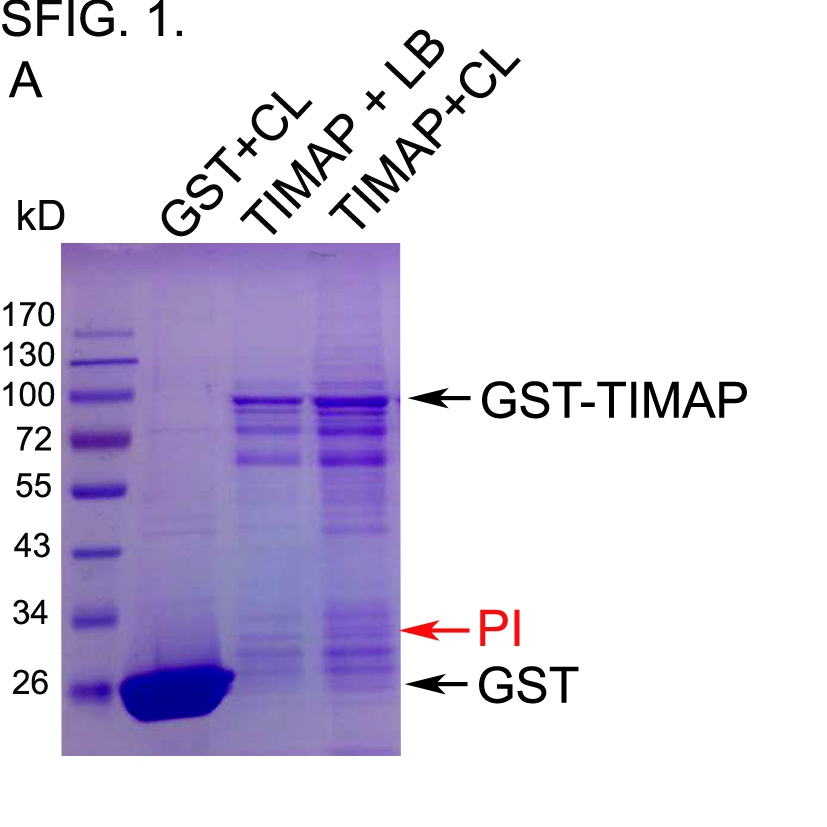

Supplement: Additional file 1 — Figure S1. Detection of TIMAP-RACK1 interaction by pull-down. Bacterially expressed glutathione S-transferase (GST) and GST-tagged wild-type TIMAP were loaded onto glutathione-Sepharose as described in Materials and Methods. After a washing step the resin samples were incubated with BPAEC lysate (CL) or cell lysis buffer (LB). Non-binding proteins were washed out and the bound proteins were eluted with 10 mM glutathion. Blue silver staining of the endothelial cell lysate (CL) and the eluted fractions after the pull-down are shown. Red arrow points the band of a possible interacting protein (PI) appearing only in the TIMAP sample incubated with EC cell lysate (TIMAP + CL). That band was cut from the gel and was identified by LC-MS/MS as RACK1 which was confirmed by Western blot (Figure 1). [file 1478-811X-11-2-S1.tiff]

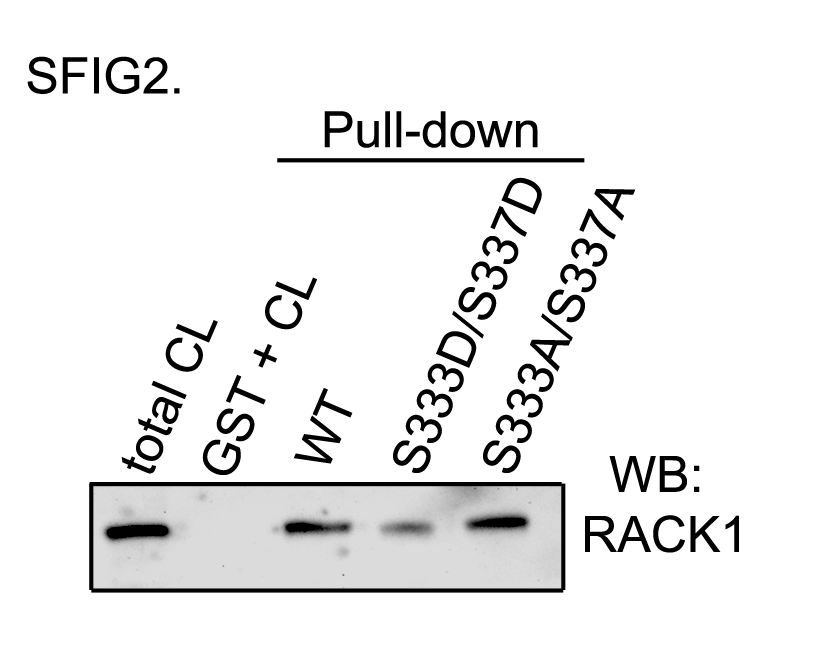

Supplement: Additional file 2 — Figure S2. Phosphomimic mutation of TIMAP attenuates RACK1 binding. GST, recombinant GST-tagged truncated wild type, S333A/S337A, and S333D/S337D mutants of a TIMAP fragment (amino acids 331–567) were loaded onto glutathione-Sepharose as described in Materials and Methods. The immobilized protein samples were incubated with BPAEC lysate. Western blot of the pull-down eluates probed with anti-RACK1 antibody is shown. CL: cell lysate. Representative data of at least 3 independent experiments are shown. [file 1478-811X-11-2-S2.tiff]

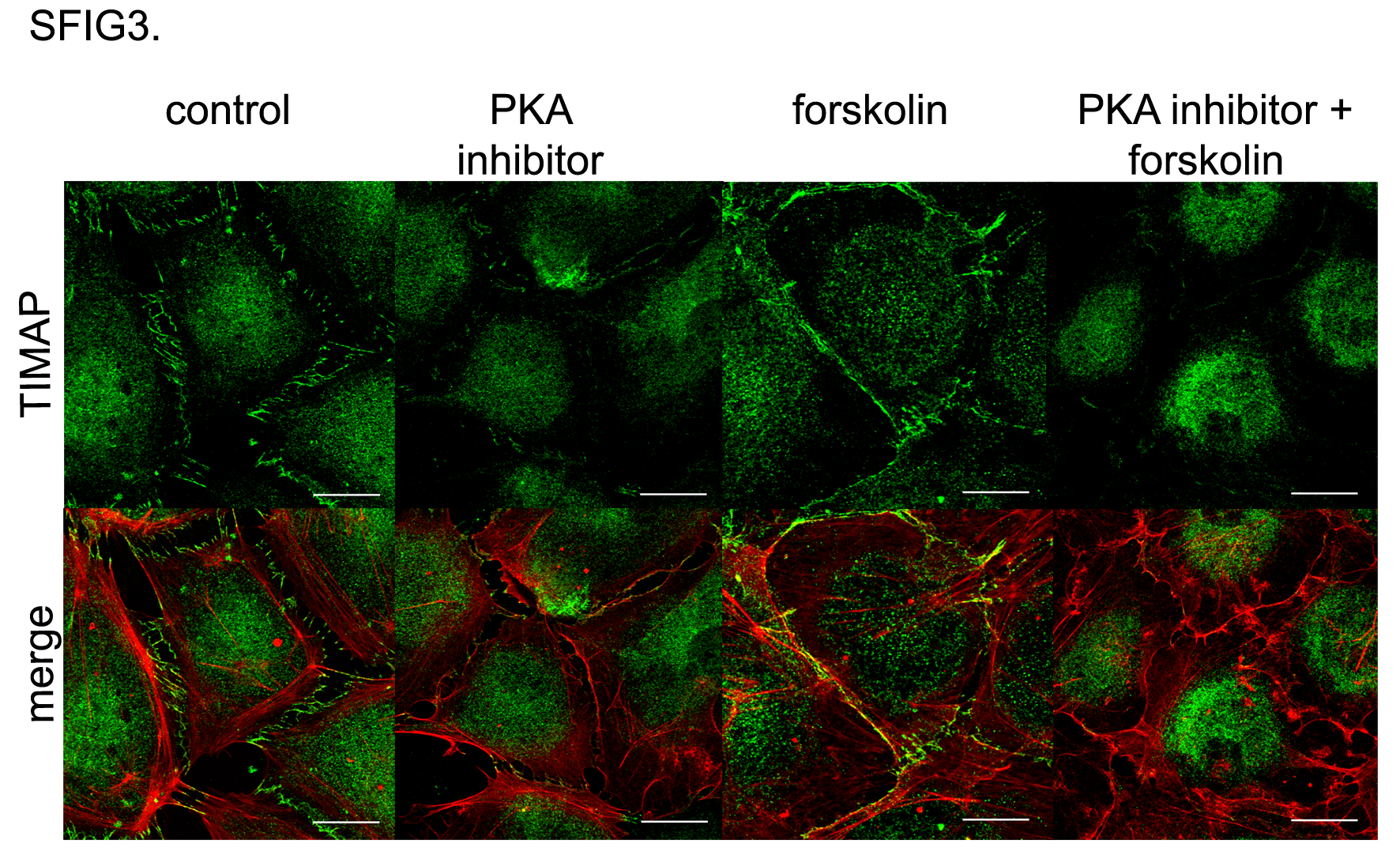

Supplement: Additional file 3 — Figure S3. Effect of PKA inhibition on the localization of TIMAP. Immunofluorescence staining of confluent HPAEC without (control), or with various treatments as follows: 10 μM H89 (PKA inhibitor) for 30 min; 50 μM forskolin for 30 min; or 10 μM H89 for 30 min followed by 50 μM forskolin for 30 min using anti-TIMAP antibody and Texas-Red Phalloidin. Scale bars: 100 μm. [file 1478-811X-11-2-S3.tiff]
